# Energy transfer from an individual silica nanoparticle to graphene quantum dots and resulting enhancement of photodetector responsivity

**DOI:** 10.1038/srep27145

**Published:** 2016-06-02

**Authors:** Sung Kim, Dong Hee Shin, Jungkil Kim, Chan Wook Jang, Soo Seok Kang, Jong Min Kim, Ju Hwan Kim, Dae Hun Lee, Jung Hyun Kim, Suk-Ho Choi, Sung Won Hwang

**Affiliations:** 1 Department of Applied Physics and Institute of Natural Sciences, College of Applied Science, Kyung Hee University, Yongin 446-701, Korea; 2 Department of Nano Science & Mechanics Engineering and Nanotechnology Research Center, Konkuk University, Chungju, Chungbuk 380-701, Korea

## Abstract

Förster resonance energy transfer (FRET), referred to as the transfer of the photon energy absorbed in donor to acceptor, has received much attention as an important physical phenomenon for its potential applications in optoelectronic devices as well as for the understanding of some biological systems. If one-atom-thick graphene is used for donor or acceptor, it can minimize the separation between donor and acceptor, thereby maximizing the FRET efficiency (*E*_FRET_). Here, we report first fabrication of a FRET system composed of silica nanoparticles (SNPs) and graphene quantum dots (GQDs) as donors and acceptors, respectively. The FRET from SNPs to GQDs with an *E*_FRET_ of ∼78% is demonstrated from excitation-dependent photoluminescence spectra and decay curves. The photodetector (PD) responsivity (R) of the FRET system at 532 nm is enhanced by 10^0^∼10^1^/10^2^∼10^3^ times under forward/reverse biases, respectively, compared to the PD containing solely GQDs. This remarkable enhancement is understood by network-like current paths formed by the GQDs on the SNPs and easy transfer of the carriers generated from the SNPs into the GQDs due to their close attachment. The R is 2∼3 times further enhanced at 325 nm by the FRET effect.

The usefulness of Förster resonance energy transfer (FRET) from one material (donor) to another material (acceptor) has been demonstrated in various kinds of coupled systems such as semiconductor quantum wells/graphene, chromophore/chromophore, nanoparticles/semiconductor, graphene/semiconductor, and molecules/graphene^1^,^2^,^3^,^4^,^5^,^6^,^7^,^8^,^9^,^10^,^11^,^12^,^13^,^14^,^15^,^16^,^17^,^18^. This nonradiative type of energy transfer occurs when donor and acceptor have states in resonance and their separation is very short, typically within only a few nanometers (0∼10 nm)^2^,^3^. The FRET process can be used to measure distances on the nanoscale^2^,^4^ and to build sensing devices by monitoring binding events^10^,^11^,^12^,^13^. In addition, graded energy structures can be engineered based on the FRET mechanism, thereby enabling cascade energy transfer^14^,^15^,^16^,^17^.

Recently, semiconductor quantum dots (SQDs) have been increasingly employed as components of nano-architectures for versatile designs providing the FRET^11^,^18^, and have proven to be valuable building blocks for light emitting devices^5^,^9^,^18^, photovoltaic cells^9^,^11^, and sensors^4^,^10^,^11^ due their unique optical properties. The flow of the energy generated in FRET structures can be optimized by the use of quantum dots (QDs) as energy donors and/or acceptors due to their tunable and narrow-emission features. Graphene quantum dots (GQDs) provide large contact area owing to their two-dimensionality, in contrast to conventional SQDs, thereby making GQDs efficient donors/acceptors. Recently, a FRET immunosensor has been developed based on the interaction between GQDs and graphene for the potential use of sensitive and selective detection of IgG^19^. GQDs typically show strong optical absorption in the near-ultraviolet (UV) region, with a tail extending out into the visible range^20^,^21^,^22^. Variously-sized GQDs with different photoluminescence (PL) colors ranging from UV to near-infrared (NIR) region have been prepared *via* various synthetic approaches^19^,^21^,^22^,^23^,^24^,^25^. Adjusting the size of GQDs is therefore expected to enhance the FRET efficiency as donors or acceptors. Especially, broad absorption spectra, long fluorescence lifetime, high photo-stability, and large quantum yield of GQDs^20^,^21^,^22^,^24^ are the additional advantages for their FRET applications in light-harvesting/sensing devices.

Silica, amorphous SiO_2_, is one of the most promising materials for nanostructured systems in view of practical requirements such as radiation hardness, stability in thermal and chemical environments, and nontoxicity. Silica is a hydrophilic material that is photophysically inert, and is not involved in energy- and electron-transfer processes because it is transparent to visible light^26^. However, the miniaturization of silica down to nanoscale introduces intrinsic peculiar properties, such as enhanced absorption and PL in broad wavelength range of NIR, visible, and UV, potentially promising for the use of silica nanoparticles (SNPs) in down-converter displays or medical nanoprobes without the need of doping with bright extrinsic fluorophores^26^,^27^,^28^,^29^. SNPs also have advantages such as easy surface modification via physical adsorption and covalent conjugation by chemical processes^26^,^28^,^29^,^30^, which could change their chemical and physical properties dramatically.

Based on these considerations, SNPs and GQDs are expected to be well employed as donors and acceptors, respectively in the FRET system, resulting in the optimized energy flow between them, promising for possible high-efficient optoelectronic devices in near UV to visible range. In this work, we propose a FRET system, composed of SNPs and GQDs, large- and small-bandgap materials, respectively, where GQDs as acceptors are attached on a single SNP as a donor, as described in Fig. 1 (and Supplementary Fig. S1). This FRET system has several novel properties; (1) Photo-excited electron-hole pairs occupy mostly the surface states of SNPs^26^,^27^,^28^,^29^, thereby making it easy for the energy to be transferred to adjacent GQDs, as shown in Fig. 1b. (2) The use of one-atom-thick material for acceptors minimizes the Förster radius, at which the FRET efficiency drops to 50%, and maximizes the contacting area of the acceptors and the donors (in strong contrast to conventional QDs), resulting in high FRET efficiency. (3) SNPs and GQDs have good chemistry due to their high-absorptive and strong-emissive properties, thereby possibly enhancing the efficiency of the optoelectronic devices based on this FRET system. (4) Both materials can be well covalently-functionalized^24^,^25^,^26^,^27^,^28^,^29^,^30^.

## Results

**Figure 2:**
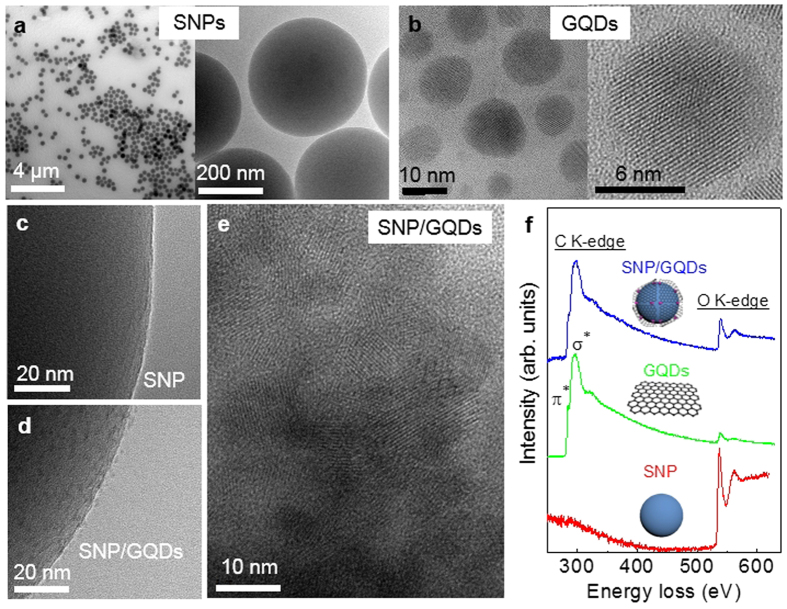
Structural characterization of SNPs, GQDs, and SNPs/GQDs hybrid. (**a**) LRTEM and HRTEM images of SNPs. The SNPs are shaped like spheres of about 300 nm average diameter. (**b**) LRTEM image of GQDs and HRTEM image of a single GQD. The GQDs of ∼12 nm size were used in the FRET system. (**c**) HRTEM image of a single SNP. (**d**) HRTEM image of a single SNP coated with GQDs. (**e**) HRTEM image of GQDs sitting on the surface of a SNP. (**f**) EELS spectra of a SNP, a GQD, and a SNP/GQDs hybrid in the C and O K-edge regions.

Figure 2a,b show low- and high-resolution transmission electron microscopy (LRTEM and HRTEM) images of SNPs and GQDs, respectively. The procedures for the synthesis of the SNPs/GQDs FRET system are in detail illustrated elsewhere (Methods and Supplementary Fig. S1). Briefly, SNPs were prepared by a modified Stöber’s method with a sol–gel process^30^,^31^,^32^. The SNPs are shaped like spheres of about 300 nm average diameter (Supplementary Fig. S2) and their surface is smooth, as shown in Fig. 2c. Ultrafine GQDs were synthesized *via* a hydrothermal method^20^,^31^ and isolated by filtering and dialysis processes. GQDs at a particular average size (*a*) were obtained by controlling the pore size of the dialysis bag through the variation of its molecular weight. Details of the fabrication processes were described in our previous report^20^. The GQDs at *a* = ∼12 nm were used in the FRET system, as shown in Fig. 2b. It was possible to see the hexagonal unit cell of GQDs in the HRTEM image^20^, demonstrating that the GQDs consist of graphene. The majority of the GQDs also proved to be composed of single-layer graphene^19^ with negligible oxygen functional groups (hydroxyl, carboxyl, and carbonyl) (Supplementary Fig. S3).

Positively-charged SNPs were dispersed in a solution of GQDs. The GQDs were then self-assembled onto the surface of the SNP shell due to strong binding interactions between the negative charged GQDs and the -NH_2_ ligands on the SNP shell, thereby producing the GQDs-coated SNP, as shown in Fig. 2d (and Supplementary Fig. S1). After the coating of the GQDs, the surface of the SNP looks corrugated and somewhat wrinkled. The HRTEM image in Fig. 2e obviously shows the GQDs sitting on the surface of the SNP. Figure 2f compares electron energy loss spectroscopy (EELS) spectra of a SNP, GQDs, and GQDs-coated SNP in the C and O K-edge regions, further confirming well formation of the SNPs/GQDs FRET system.

**Figure 3:**
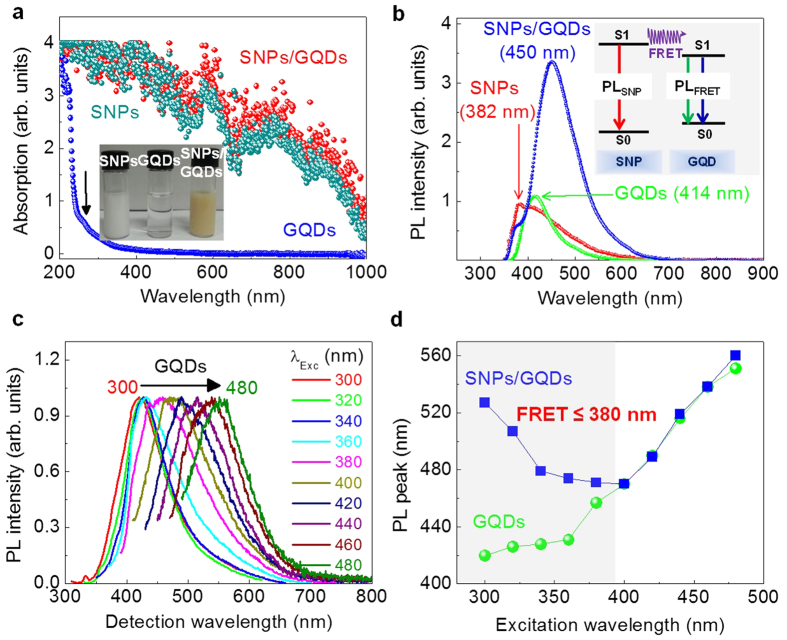
Absorption and PL spectra of SNPs, GQDs, and SNPs/GQDs hybrid. (**a**) Absorption spectra of SNPs, GQDs, and SNPs/GQDs hybrid. The inset shows photographs of the three samples emitting light with different colors under ambient condition. (**b**) PL spectra of SNPs, GQDs, and SNPs/GQDs hybrid, excited at 325 nm. The inset describes the FRET effect responsible for the PL of SNPs/GQDs hybrid. (**c**) Excitation-wavelength-dependent PL spectra of GQDs, measured at 414 nm. (**d**) Excitation-wavelength-dependent PL peak shifts of GQDs and SNPs/GQDs hybrid. No energy transfer occurs from SNPs to GQDs at λ > 380 nm, but for ≤380 nm, the peak wavelength of SNPs/GQDs hybrid increases with decreasing λ, indicating the energy transfer from SNPs to GQDs.

The optical properties of the individual donors and acceptors are important for the analysis of the FRET system. Absorption spectra of GQDs, SNPs, SNPs/GQDs hybrid in deionized (DI) water are shown in Fig. 3a. The inset in Fig. 3(a) shows photographs of the three samples emitting light with different colors under ambient condition. In the absorption spectrum of GQDs, a shoulder is observed at ∼267 nm, originating from the intrinsic states of GQDs^21^,^22^. On the other hand, the SNPs show a broad absorption spectrum with the intensity gradually decreasing up to 1000 nm, resulting from the defect states at the surface of SNPs^26^,^28^,^29^,^30^. The absorption spectrum of SNPs/GQDs hybrid is slightly up-shifted with respect to that of SNPs, possibly resulting from the superposition of the absorption intensities by GQDs and SNPs.

Figure 3b shows PL spectra of SNPs, GQDs, and SNPs/GQDs hybrid, excited by a 325 nm laser line. The major PL peak of SNPs at around 382 nm is known to originate from the defect states at the shell (or the surface) of SNPs^26^,^27^,^28^,^29^. Especially, the PL spectrum of SNPs ranges widely from 350 to 700 nm (UV to visible), very promising for the use of SNPs as donors in the FRET system. The PL spectrum of the GQDs shows a strong peak at 414 nm with a Stokes shift of 147 nm (1.65 eV), compared to the absorption band at 267 nm, as shown in Fig. 3a. The PL spectrum of SNPs/GQDs hybrid exhibits a major emission band at ∼450 nm, which is attributed to the excitonic band-edge emission of GQDs, with a SNPs-related PL shoulder at ∼382 nm. The apparent redshift of about 36 nm possibly results from new states coherently superposed from individual GQD wave functions, led by delocalized electronic excitations in strongly-coupled GQDs on SNPs, as similarly explained in several FRET systems composed of SQDs^33^,^34^,^35^, showing such PL redshift.

Excitation-dependent PL experiments were done to demonstrate the FRET effect of the SNPs/GQDs hybrid. By the excitation at wavelengths (λ) from 300 to 480 nm, the PL peak of GQDs is shifted from 421 to 551 nm, as shown in Fig. 3c. In the previous intensive studies, the excitation-dependent PL behaviors of GQDs have been attributed to the optical selection of differently-sized GQDs and their edge states^20^,^22^. As summarized in Fig. 3d, the peak wavelength of SNPs/GQDs hybrid decreases as λ decreases from 480 to 400 nm (Supplementary Fig. S4). This λ-dependent PL-peak shifts are nearly identical to those of GQDs, indicating no energy transfer from SNPs to GQDs at λ ≥ ∼400 nm because the electron-hole pairs responsible for the PL peaked at 382 nm cannot be produced in SNPs. However, for λ < ∼400 nm (≤380 nm, exactly in this work), the peak wavelength of SNPs/GQDs hybrid increases with decreasing λ (Supplementary Fig. S4), indicating that the energy transfer occurs from SNPs to GQDs below ∼400 nm.

**Figure 4:**
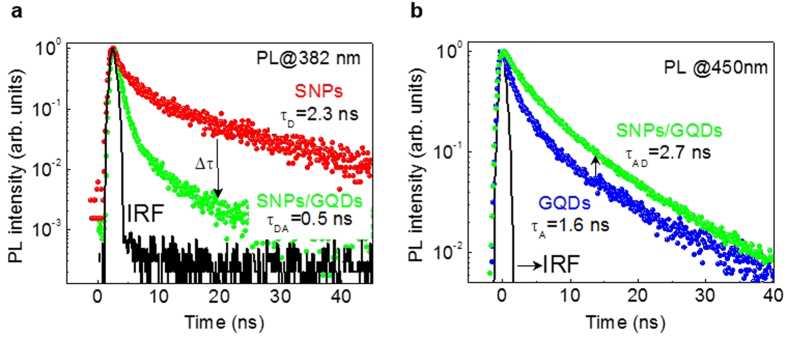
PL decay curves of SNPs, GQDs, and SNPs/GQDs hybrid. (**a**) Decay curves of the donor emission at 382 nm for SNPs and SNPs/GQDs hybrid. The average PL lifetime reduces from ∼2.3 to ∼0.5 ns. (**b**) Decay curves of the acceptor emission at 450 nm for GQDs and SNPs/GQDs hybrid. The average PL lifetime increases from ∼1.6 to ∼2.7 ns. The FRET effect is evidenced by the shortening of the donor decay accompanied by the lengthening of the acceptor decay. The excitations were done at 305 nm.

Figure 4a shows decay curves of the donor emission at 382 nm for SNPs (donors) and SNPs/GQDs hybrid, excited at a wavelength of 305 nm. The average PL lifetime is reduced from ∼2.3 ns in the SNPs to ∼0.5 ns in the hybrid, where 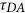 = ∼0.5 ns and 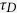 = ∼2.3 ns are defined as the PL lifetimes of the donor in the presence and absence of the acceptor, respectively. Figure 4b shows decay curves of the acceptor emission at 450 nm for GQDs (acceptors) and SNPs/GQDs hybrid, also excited at a wavelength of 305 nm. Due to the energy transfer, the average lifetime (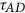 = ∼2.7 ns) of the hybrid structure is larger than that (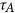 = ∼1.6 ns) of GQDs. The carriers that are photo-excited but disappear by non-radiative recombination in SNPs should be also considered as another factor for the increase of the acceptor-emission lifetime because some of them can be transferred to GQDs before recombination. This long-term storage of energy is not visible in the decay curves of SNPs, as shown in Fig. 4a, but after the transfer of the photo-excited carriers to neighboring GQDs, the excitations can be visible through the PL emission from GQDs (so-called trapped-exciton-recycling effect^14^,^15^). The simultaneous occurrence of shortening of the donor decay and lengthening of the acceptor decay provides strong evidence of the energy transfer.

**Figure 5:**
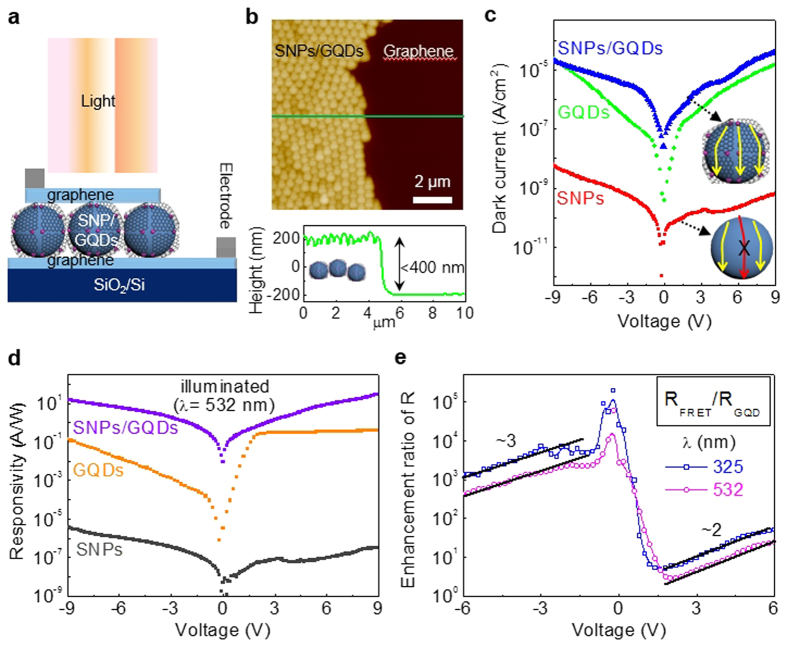
A schematic of the FRET system-based PD and its photoresponse. (**a**) A schematic of PD structure composed of the FRET system sandwiched between single-layer graphene sheets. (**b**) AFM image and height profile of SNPs/GQDs hybrid on graphene. (**c**) Dark I-V curves of SNPs, GQDs, and SNPs/GQDs hybrid. The inset describes the current paths on a SNP and a SNP/GQDs hybrid. (**d**) Responsivities of SNPs, GQDs, and SNPs/GQDs hybrid as functions of bias voltage, excited at 532 nm. (**e**) Enhancement ratios of the responsivities (R_FRET_/R_GQD_) at 325 and 532 nm as functions of bias voltage.

In our previous report^23^, high photo-responsivity (0.2∼0.5 A/W) was achieved in the broad spectral range of UV to NIR from PDs consisting of multiple-layer GQDs sandwiched between graphene sheets. Figure 5a shows a schematic diagram of a typical PD structure of SNPs/GQDs FRET system, composed of SNPs/GQDs hybrid sandwiched between single-layer graphene sheets, which is named as FRET-PD. The SNPs/GQDs hybrid is nearly single layer, judging from the total thickness below 400 nm, estimated from the atomic force microscopy (AFM) image and height profile, as shown in Fig. 5b, if random and slightly-fluctuated orientations of the SNPs/GQDs are considered. The graphene sheets proved to be slightly p-type and single layer by several analysis tools (Supplementary Fig. S5). The control samples were also fabricated from the same PD structure containing solely SNPs or GQDs, named as SNP-PD or GQD-PD, respectively. The SNPs, GQDs, and SNPs/GQDs hybrid used in the PDs produced PL spectra similar to those of the materials in DI water, demonstrating that each material well exists in the PD structure and experiences almost no structural alterations during the PD fabrication.

Figure 5c shows dark current (DC)-voltage (I-V) curves of SNP-PD, GQD-PD, and FRET-PD. Positive voltages were applied to the Ag electrode on the upper graphene layer with respect to that on the lower graphene layer/SiO_2_ under forward bias. The dark I-V curve of the SNP-PD is asymmetric and shows very-low leakage current under forward bias as well as under reverse bias. These leakage currents can be attributed to flow of carriers through the available density of surface states of SNPs between the metallic graphene sheets^36^,^37^,^38^, as shown in the inset of Fig. 5c. The dark I-V curve of the GQD-PD exhibits asymmetric and nonlinear properties with varying bias voltage, as shown in Fig. 5c, based on tunneling of carriers through the available density of states of GQDs between the graphene sheets, as explained in our previous report^23^. The FRET-PD shows similar dark I-V characteristics. The current density of the FRET-PD is maximally ∼10^5^/10^4^ and ∼5/21 times larger than those of the SNP-PD and GQD-PD, respectively under forward/reverse biases. The large enhancement of DC in the FRET-PD possibly results from the following reasons; (1) Network-like current paths are formed by the GQDs on the SNPs in the FRET-PD, as shown in the inset of Fig. 5c, (2) The carriers generated from the surface states of the SNPs can be easily transferred to the GQDs under the applied electric field because the SNPs are very closely attached with the GQDs. This kind of transfer behavior was already observed/analyzed in the graphene sheet on SiO_2_, as reported before^36^,^37^, (3) Unexpected sources of leakage can be developed during the device fabrication, but should be negligible, judging from the extremely-low DC level of the SNP-PD despite the same fabrication process.

## Discussion

The energy transfer mechanism can be studied in more detail by analyzing time-resolved PL decays of SNPs (donors), GQDs (acceptors), and their hybrid. As shown in Fig. 4, the PL decay curves contain fast and slow components, and can be therefore expressed by a two-exponential function, 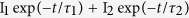, where τ_1_ and τ_2_ are the fast and slow decay times, respectively, and I_1_ and I_2_ are the contributions of the corresponding parts to the total PL intensity^5^,^11^. The average decay lifetime, τ is calculated as the intensity-weighted means, as given in the following equation^4^,^11^:


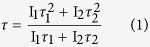


The time-resolved data can be also used to calculate the *E*_FRET_ using the following equation^4^,^11^:


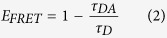


This results in an *E*_FRET_ of ∼78% from the data of 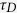 = ∼2.3 ns and 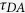 = ∼0.5 ns, as obtained in Fig. 4a.

To quantify the functioning of the FRET system in its photoactivity, we compared the PD performances under illuminations at λ < 400 nm and at λ > 400 nm. First, a light with a wavelength of 532 nm (power = 3 mW/cm^2^) was used for the photoexcitation to exclude the FRET effect. The photocurrent (PC)/DC ratios of the SNP-PD are ∼2.4 and ∼3.4 at ±5 V, respectively (Supplementary Fig. S6), resulting in very low R of ∼7 × 10^−8^ and ∼8 × 10^−7^ A/W at ±5 V, respectively, as shown in Fig. 5d, originating from the photo-carriers excited from the defect states at the surface of SNPs, as discussed in the absorption spectrum of Fig. 3a. In contrast, the responsivity of the GQD-PD is much larger (∼0.31 and ∼0.007 A/W at ±5 V, respectively), as shown in Fig. 5d, resulting from the photoexcitation of electron-hole pairs in GQDs, as proved in our previous report^23^. The FRET-PD shows even larger responsivity under forward bias as well as under reverse bias, compared to the SNP-PD and GQD-PD. Figure 5e shows the ratio of the responsivities of the FRET-PD and the GQD-PD (R_FRET_/R_GQD_). The R_FRET_/R_GQD_ is about 10^0^∼10^1^ and 10^2^∼10^3^ under forward and reverse biases, respectively, and the R_FRET_ is 0.93∼4.14 A/W at −2∼−5 V, as shown in 5d, much larger than those reported for commercial Si and InGaAs PDs (∼0.5 and ∼0.9 A/W, respectively)^36^,^37^. This remarkable enhancement of R in the FRET-PD despite no FRET effect at λ = 532 nm (>400 nm) can be understood based on the same reasons (1) and (2) for the photoconduction of the carriers, as discussed above for the large DC enhancement.

Secondly, to see the FRET effect, the photoexcitation was done at λ = 325 nm (< 400 nm) (same power). The PC and R of the SNP-PD at λ = 325 are a bit enhanced compared to those at λ = 532 nm (Supplementary Figs S6 and S7), resulting from higher absorption at 325 nm, as shown in Fig. 3a. The PC and R of the FRET-PD are much larger than those of the SNP-PD as well as those of the GQD-PD even at λ = 325 nm (Supplementary Figs S6 and S7), similar to the case at λ = 532 nm, as shown above. The R_FRET_/R_GQD_ values at λ = 325 nm is ∼2 and ∼3 times larger under forward and reverse biases, respectively than those at λ = 532 nm, as shown in Fig. 5e, resulting from the FRET effect.

These results demonstrate that the SNPs/GQDs hybrid is a highly-efficient FRET system with an *E*_FRET_ of ∼78%. The GQDs facilitate the transport of carriers in the hybrid due to the unique two-dimensionality of graphene. The achievement of the extremely-high photoresponse from the FRET-PD implies that the SNPs/GQDs FRET system can be used as a building block for a variety of possible transparent and flexible optoelectronics applications.

## Methods

### Preparation of graphene quantum dots (GQDs)

GQDs were fabricated by the following processes. Graphene oxide (GO) sheets were obtained from natural graphite powder by a modified Hummers method^20^,^39^. The GO sheets were then deoxidized in a tube furnace at 250 °C for 2 h under Ar ambient to prepare micrometer-sized graphene sheets (GSs). The size of the GSs was reduced by repeated processes of oxidation/reduction, and finally, ultrafine GQDs were isolated by filtering and dialysis processes. Nanoporous membranes with 20- and 200-nm pores were used in the filtering processes. GQDs at a particular size were obtained by controlling the pore size of the dialysis bag through the variation of its molecular weight. Details of the processes are described elsewhere^20^.

### Preparation of silica nanoparticles (SNPs)

The SNPs were fabricated based on a modified Stöber method^26^,^30^, in which it is possible to obtain highly monodisperse SNPs with a diameter ranging from 15 to 800 nm by maintaining careful control of the reaction conditions. The major solutions used to synthesize SNPs were Tetraethylorthosilicate (TEOS) (98%, Sigma Aldrich), absolute ethanol (99.5%, Systerm), and ammonium hydroxide (NH_3_ 25∼30%, Merck). Deionized (DI) water was used at every stage of reaction and washing. Solvent, ammonium hydroxide, and DI water were first mixed thoroughly, and TEOS was then added rapidly. The size of SNPs was controlled by varying the amounts of reactants (TEOS and DI water) and catalyst (ammonium hydroxide), and changing the stirring speed. The reaction was done at 25 °C for 1 h. The average size of the SNPs was determined by transmission electron microscopy.

### Fabrication of SNPs/GQDs FRET system

SNPs/GQDs FRET system was fabricated by simply mixing positively-charged SNP-NH_2_ particles and negatively-charged GQDs dispersion^40^,^41^. SNPs were first mixed with 3 M HCl, and the slurry was left overnight. The next day, it was filtered and dried at 100 °C in an oven for 3 h. Acid-treated SNPs and GQDs were added to a three-necked flask with an ultrasonic treatment while the pH was adjusted to 10 with dilute ammonia. By strong binding interactions between the negative-charged GQDs and the −NH_2_ ligands on the shell of SNTs, the SNPs/GQDs FRET system was produced.

### Fabrication of photodetectors (PDs)

First, the single-layer graphene prepared by chemical vapour deposition was transferred to 300 nm SiO_2_/n-type Si wafers, and annealed at 400 °C for 1 h in vacuum to remove the surface adsobates. A 200-μl solution of SNPs (or GQD, GQDs/SNPs) was then dropped and spin-coated on the 10 × 10 mm^2^ graphene/SiO_2_/n-type Si wafer, and annealed at 100 °C for 1 min. Subsequently, a 5 × 5 mm^2^ single-layer graphene was transferred on ∼1/4 area of the SNPs/graphene/SiO_2_/n-type Si wafer, and annealed at 400 °C for 1 h in vacuum. As a result, the graphene/SNPs/graphene sandwich structure was formed on the ∼1/4 area of the SiO_2_/n-type Si wafer. Ag electrodes of 1 mm diameter and 1 μm thickness were deposited on the top of both graphene sheets to complete the SNP (or GQDs, GQDs/SNPs) PD devices.

### Device characterization

Current-voltage (I-V) measurements to characterize the electrical behaviors of the PDs were carried out using a Keithley 2400 source meter controlled by a LabView program. During the measurements, the PDs were mounted in a dark, electrically-shielded, and optically-sealed chamber on the optical table to reduce vibrational noise. To quantify the photoresponse of the FRET system, we compared the PD performances under illuminations at λ = 325 nm and at λ = 532 nm, respectively by focusing the light onto the PD devices with a spot size of ∼5 × 5 mm^2^. The power density of the incident light on the sample surface was 3 mW/cm^2^. The light was modulated with a mechanical chopper (Standard Research Systems) with a frequency of 30 Hz and the PC response at various bias voltages was recorded with a Keithley 2400 source meter.

## Additional Information

**How to cite this article**: Kim, S. *et al.* Energy transfer from an individual silica nanoparticle to graphene quantum dots and resulting enhancement of photodetector responsivity. *Sci. Rep.* **6**, 27145; doi: 10.1038/srep27145 (2016).

## Supplementary Material

Supplementary Information

## Figures and Tables

**Figure 1:**
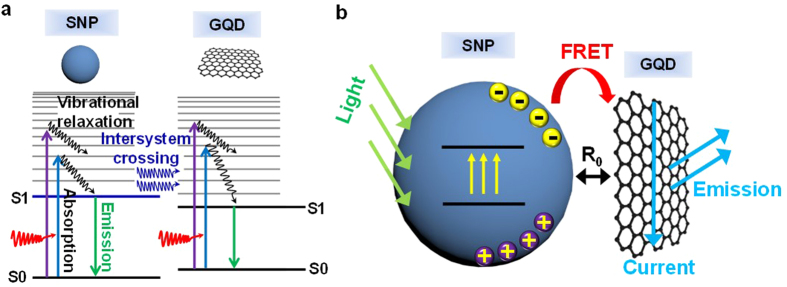
Schematic diagrams describing the FRET system. (**a**) Band structures of SNP and GQD. Possible absorption/emission/transfer processes are described. Here, S0 and S1 indicate ground and lowest excited states, respectively. SNPs are highly absorptive, especially in the range of the emission spectrum of GQDs. Differently-colored PL of variously-sized GQDs ranges from UV to near-infrared region. (**b**) A schematic of a typical FRET system composed of SNPs and GQDs as donors and acceptors, respectively. Photo-excited electron-hole pairs occupy mostly the surface states of SNPs, thereby making it easy to transfer energy to adjacent GQDs. The distance at which the FRET efficiency drops to 50% is defined as the Förster radius (R_0_), typically in the range of 1–10 nm. The use of one-atom-thick GQDs for acceptors can minimize R_0_ and maximize the contacting area of the acceptors and the donors.
