# Supplementary material for: Energy transfer from an individual silica nanoparticle to graphene quantum dots and resulting enhancement of photodetector responsivity
Source: Sci Rep. 2016 Jun 2;6:27145. doi: 10.1038/srep27145 (PMC4889998; doi:10.1038/srep27145)
Supplement: Supplementary Information [file srep27145-s1.doc]

**Supplementary Information**

**for**

**Energy transfer from an individual silica nanoparticle to graphene quantum dots and resulting enhancement of photodetector responsivity**

Sung Kim,^1^ Dong Hee Shin,^1^ Jungkil Kim,^1^ Chan Wook Jang,^1^ Soo Seok Kang,^1^ Jong Min Kim,^1^ Ju Hwan Kim, Dae Hun Lee,^1^ Jung Hyun Kim,^1^ Suk-Ho Choi,^1†^ Sung Won Hwang,^2^

***^1^*** Department of Applied Physics and Institute of Natural Sciences, Kyung Hee University, Yongin 446-701, Korea

**^2^** Department of Nano Science & Mechanics Engineering and Nanotechnology Research Center, Konkuk University, Chungju, Chungbuk 380-701, Korea

_____________________________________________________________

**^†^**To whom correspondence should be addressed. E-mail: sukho@khu.ac.kr

**METHODs**

**Graphene growth**

Graphene layers were grown on 70-μm-thick Cu foils (Wacopa, 99.8 purity) in a graphite-heater-based chemical-vapor-deposition (CVD) quartz tube furnace at a growth temperature of 1000 ^o^C with 10-sccm H_2_ and 20-sccm CH_4_ flowing at a pressure of 3 Torr. The graphene/Cu stack was spin-coated with poly(methyl methacrylate) (PMMA), and the Cu was then etched in a 1 M ammonium persulfate for 10 h. The graphene/PMMA stack was then placed in DI water before transferring to the 300 nm SiO_2_/n-type Si wafers and blow-dried with dry N_2_. The PMMA/graphene/SiO_2_/n-Si stack was then heated on a hot plate in air at 180 ^o^C for 2 h to cure the PMMA. After the samples were cooled to room temperature (RT), the PMMA was stripped by soaking them in acetone for 1 h at RT. Subsequently, the graphene/SiO_2_/n-Si stack was put in isopropyl alcohol for 10 min and dried by blowing N_2_ to minimize the water traps possibly present at the graphene/SiO_2_ interface, and annealed at 400 ^o^C for 1 h in vacuum to remove the surface adsorbates.

**Structural and optical characterization**

Morphologies of samples were analyzed using a transmission electron microscope (200 kV, JEM-2100F, Jeol Ltd., Japan) with electron energy loss spectroscope (EELS). To make the TEM/EELS specimens the samples were dispersed in DI water, a drop of which was then put on a SiO-coated Cu grid (Tedpella Inc.). Topographic image and height profile of SNPs/GQDs FRET system were obtained in a non-contact mode of atomic force microscope (Park system, model XE-100). Raman-scattering behaviors excited using a 532 nm laser diode was employed to inspect the number and quality of graphene layer.

Transmittance and absorption spectra were measured using an Agilent 8453 UV-visible Spectrophotometer. The excitation sources were ~40 mW/cm^2^ tungsten/deuterium lamps from which monochromatic light was selected using a grating monochromator and associated filters. Photoluminescence (PL) spectra were measured at RT using the 325 nm line of a HeCd laser as the excitation source. Emitted light was collected by a lens and analyzed using a grating monochromator and a GaAs photomultiplier tube. Standard lock-in detection techniques were used to maximize the signal-to-noise ratio. Excitation-dependent PL spectra were measured in a PTI's QuantaMaster^TM^ 30 System at RT by varying the excitation wavelength from a 450 mW Xenon arc lamp. Time-resolved PL (TRPL) measurements were performed at an excitation wavelength of 305 nm with a pulse repetition rate of 500 KHz by using frequency-doubled, mode-locked (150 fs), Ti:sapphire laser system as an excitation source and a time-correlated single photon counting system for detection. The laser instrument response function (IRF) was about 18 ps. The plastic cuvettes used for the optical measurements covered the transmission wavelength range of 280~800 nm.

**Fabrication of graphene field effect transistors (GFETs)**

GFETs were fabricated through photolithography patterning procedures by using the transferred graphene. First, source and drain electrodes of 200/200 µm in width/length were patterned by photolithography followed by electron beam evaporation of Cr/Au bilayer with thicknesses of 5/30 nm and standard lift-off process. The graphene channel of 100 µm length was then patterned by another photolithography and subsequent O_2_-plasma etching. The fabricated GFETs were heated in a rapid thermal annealing apparatus at 250 °C for 10 min under N_2_ ambient to eliminate residues on graphene layers in GFETs.

**Figures for Supplementary Information**

**Figure S1.** Summary of the procedures for fabricating the FRET system composed of SNPs and GQDs and high-resolution TEM images of GQDs, a SNP, and SNP/GQDs hybrid.

**Figure S2.** Scanning electron microscopy image of SNPs.

**Figure S3.** (a) X-ray photoelectron spectroscopy and (b) EELS spectra of GQDs.

**Figure S4.** Excitation-wavelength-dependent PL spectra of SNPs/GQDs hybrid.

**Figure S5.** (a) Transmittance, (b) Raman spectrum, and (c) Dirac curve of graphene used in this work.

**Figure S6.** Dark and photo I-V curves of (a) SNPs, (b) GQDs, and (c) SNPs/GQDs hybrid. The photo-excitations were done at 325 and 532 nm.

**Figure S7.** Photo-responsivities of SNPs, GQDs, and SNPs/GQDs hybrid as functions of bias voltage, excited at 325 nm.
